# Targeting folate receptor β positive tumor-associated macrophages in lung cancer with a folate-modified liposomal complex

**DOI:** 10.1038/s41392-020-0115-0

**Published:** 2020-01-22

**Authors:** Yan Tie, Heng Zheng, Zhiyao He, Jingyun Yang, Bin Shao, Li Liu, Min Luo, Xia Yuan, Yu Liu, Xiangxian Zhang, Hongyi Li, Min Wu, Xiawei Wei

**Affiliations:** 1 Laboratory of Aging Research and Cancer Drug Target, State Key Laboratory of Biotherapy and Cancer Center, National Clinical Research Center for Geriatrics, West China Hospital, Sichuan University, Chengdu, 610041 Sichuan PR China; 2 Department of Oncology, Sichuan Cancer Hospital and Institute, Sichuan Cancer Center, School of Medicine, University of Electronic Science and Technology of China, Chengdu, 610041 Sichuan PR China; 3 Department of Gynecology and Obstetrics, Key Laboratory of Birth Defects and Related Diseases of Women and Children, Ministry of Education, West China Second Hospital, Sichuan University, Chengdu, 610041 Sichuan PR China; 4 Department of Biomedical Sciences, School of Medicine and Health Sciences, University of North Dakota, Grand Forks, ND 58202 USA

**Keywords:** Lung cancer, Prognostic markers

## Abstract

Tumor-associated macrophages (TAMs) facilitate cancer progression by promoting tumor invasion, angiogenesis, metastasis, inflammatory responses, and immunosuppression. Folate receptor β (FRβ) is overexpressed in TAMs. However, the clinical significance of FRβ-positive macrophages in lung cancer remains poorly understood. In this study, we verified that FRβ overexpression in lung cancer TAMs was associated with poor prognosis. We utilized a folate-modified lipoplex comprising a folate-modified liposome (F-PLP) delivering a BIM-S plasmid to target both lung cancer cells and FRβ-positive macrophages in the tumor microenvironment. Transfection of LL/2 cells and MH-S cells with F-PLP/pBIM induced cell apoptosis. Injection of F-PLP/pBIM into LL/2 and A549 lung cancer models significantly depleted FRβ-positive macrophages and reduced tumor growth. Treatment of tumor-bearing mice with F-PLP/pBIM significantly inhibited tumor growth in vivo by inducing tumor cell and macrophage apoptosis, reducing tumor proliferation, and inhibiting tumor angiogenesis. In addition, a preliminary safety evaluation demonstrated a good safety profile of F-PLP/pBIM as a gene therapy administered intravenously. This work describes a novel application of lipoplexes in lung cancer targeted therapy that influences the tumor microenvironment by targeting TAMs.

## Introduction

Lung cancer is the most commonly diagnosed cancer and is the leading cause of cancer deaths among males and females worldwide.^1^ According to global cancer statistics, ~1.8 million new lung cancer cases occur per year.^1^ The 5-year survival rate ranges from 6 to 14% in male lung cancer patients and from 7 to 18% in female patients.^2^ Molecular targeted therapy for lung cancer has been an important and intense research field over the last several years.^3^ The most widely studied types of targeted therapies are to block overexpressed genes, increase activation of tumor suppressor genes, target tumor cell antigens, induce immunity against tumor cell antigens, or inhibit or alter the signals that control the growth and survival of tumor cells.^3^ Most current targeted cancer therapies focus on killing tumor cells, but the immunosuppressive impact of the tumor microenvironment is not negligible.

Tumor-associated macrophages (TAMs), which are local tumor macrophages, account for the majority of leukocytes in the tumor microenvironment. Once mononuclear macrophages are recruited to the tumor microenvironment, they polarize into M1 or M2 macrophages affected by various factors. M1 macrophages exert antitumor effects through the overexpression of cytokines such as IL-1 and IL-6. M2 macrophages promote tumor invasion, metastasis, and inflammatory responses. TAMs in the tumor microenvironment tend to adopt the anti-inflammatory M2-like phenotype.^4^ TAMs participate not only in tumor invasion, growth, angiogenesis, metastasis, and immunosuppression but also in neovascularization and matrix degradation.^5^ Strategies that target TAMs aim to reduce the quantity and change the function of TAMs. The prognosis of lung cancer patients is closely related to TAMs.^5,6^ However, the clinical significance of FRβ-positive macrophages in lung cancer remains poorly understood.

The folate receptor (FR) family consists of four members, including FRα (or FOLR1), FRβ (or FOLR2), FRγ (or FOLR3) and FRδ (or FOLR4). FRα and FRβ, which are attached to the cell membrane through glycosylphosphatidylinositol (GPI) anchors, are overexpressed in tumor cells and TAMs, respectively.^7–11^ FRγ is secreted from tissues of hematopoietic origin due to the lack of an efficient signal for GPI modification and has a much lower affinity for folate than FRα and FRβ.^12–14^ FRδ has proven elusive in human tissues, suggesting that it is highly restricted both spatially and temporally.^14,15^ Currently, clinical studies on FRα-conjugated chemotherapeutics and humanized anti-FRα antibodies are being evaluated.^16,17^ FRβ has been demonstrated to be overexpressed in M2-polarized TAMs, which reveals the potential therapeutic value of folate-drug conjugates.^7,18–20^ Injection of a recombinant immunotoxin consisting of the immunoglobulin heavy and light chain Fv fragments of an anti-mouse FRβ antibody and a 38-kDa portion of *Pseudomonas* exotoxin. A significantly depleted TAMs and reduced tumor growth in an experimental glioma model.^21^ Depletion of TAMs by zoledronic acid entrapped in folate-linked liposomes can selectively induce in vitro cytotoxicity via FRs.^22^ All these results reveal that FRβ is an attractive target for TAM-selective delivery, but no FRβ-associated targeted therapy for lung cancer TAMs has been reported.

Gene therapy against lung cancer has been reported to have potential efficacy and has been a worldwide research field over the last two decades.^23^ Among the investigated genes, those in the BCL-2 family play a crucial role in lung cancer treatments that depend on mitochondria-mediated apoptosis.^24^ In this family, all members contain at least one of four BCL-2 homology (BH) domains, named BH1 to BH4.^25^ BIM (BCL-2-interacting mediator of cell death), one of the BH3-only subfamily members, has many isoforms that encode proteins that bind to BCL-2, including BIM-EL (variant 1), BIM-L (variant 6), and BIM-S (variant 11).^26^ Moreover, the proapoptotic protein BIM has been demonstrated to be a key modulator of apoptosis following effective targeted therapy, and deficiencies in BIM expression result in targeted therapy resistance.^27^ BIM-S has been reported to be the most potent isoform in inducing apoptosis, but research on BIM-S is still rare.^26^

Therefore, M2 macrophages promote tumor progression through multiple pathways. Targeting M2 macrophages to treat cancers may achieve a promising therapeutic outcome. However, a few specific receptor types expressed on macrophages can be used for targeted therapy by drug-loaded nanoparticles. Identification of the specific receptor types expressed on TAMs is impending and crucial. Recent studies revealed that macrophages had a high level of FRβ expression. FRβ might be an ideal target for macrophage-related therapy. Therefore, we utilized a folate-modified lipoplex comprising a folate-modified liposome (F-PLP) delivering a BIM-S plasmid (pBIM) to target lung cancer cells and focused on the efficacy of therapies targeting macrophages in the tumor microenvironment.

## Materials and methods

### Materials and preparation and characterization of FR-targeting liposomes and lipoplexes

MPEG-succinyl-cholesterol conjugate (mPEG-suc-Chol) and folate-PEG-succinyl-cholesterol conjugate (F-PEG-suc-Chol) were synthesized and purified by our laboratory as previously described.^28,29^ A pBIM was used as described in our previous research.^30^ The vector carrying BIM-S was pVAX1, and the selected insertion site was NheI/XhoI. The sequence was generated by OriGene (MC208191, USA). The NCBI reference serial number is NM_009754.3. The pVAX vector and glucose injection (5%) were used as negative controls. We used an eGFP (enhanced green fluorescent protein) plasmid for transfection in vitro for fluorescence imaging and flow cytometry analysis. We extracted the BIM plasmid and pVAX vector according to the instructions of the EndoFree Plasmid Purification Kit (Qiagen, Germany).

F-PLPs were prepared with a film dispersion method, as described previously, with DOTAP, Chol, mPEG-suc-Chol, and F-PEG-suc-Chol.^31^ The procedure was the same as that described in our previous report.^32^ FR-targeting lipoplexes were prepared according to the methods described in our previous report; F-PLP was mixed with pBIM or pVAX for 30 min at room temperature to formulate F-PLP/pBIM or F-PLP/pVAX, respectively. All experiments were performed in triplicate.

After the lipoplexes were prepared, 1% (w/v) agarose gel (Invitrogen, USA) electrophoresis was conducted in pH 7.4 TAE buffer (40 mM Tris/HCl, 1% acetic acid, 1 mM EDTA) containing the nucleic acid stain GoldView at a constant voltage of 120 V for 25 min at room temperature to determine the optimal proportion between F-PLP and pBIM. We visualized and digitally photographed the electrophoresis gels with a gel documentation system (Gel Doc 1000, Bio-Rad, USA).

The particle size and zeta potential of the lipoplexes and liposomes were determined using a Zetasizer Nano ZS ZEN 3600 instrument (Malvern, UK). All results are the mean of three test runs.

### Identification of the expression of FRα and FRβ in lung cancer

Before data extraction and tissue microarray construction, ethical approval was obtained from the ethics committee of Shanghai Outdo Biotech Co., Ltd. In this study, 184 patients who were diagnosed with primary lung squamous cell carcinoma and lung adenocarcinoma from 2004 to 2009 were selected. Clinical and pathological data were extracted from surgical pathology and medical records. The histological names followed the World Health Organization classification, and the staging was based on the 8th edition of the American Joint Committee on Cancer (AJCC).

Tumor tissues were fixed with formalin, embedded in paraffin, and arranged into tissue chips. Tissue chips were placed in an oven, and the wax was baked for 1 h at 63 °C. The tissue chips were dewaxed with xylene and dehydrated with gradient alcohol. Citric acid solution (pH = 6.0) was used for high-pressure repair for 5 min. The tissue chips were incubated with serum for 20 min after blocking with 3% H_2_O_2_ for 20 min. The FRα primary antibody (1:100, SIG-3619, BioLegend, USA) and FRβ primary antibody (1:50, SAB1307181, Sigma-Aldrich, USA) were added dropwise and incubated at 4 °C overnight. Enzyme-labeled secondary antibody was added dropwise, and the reaction was placed at room temperature for 30 min. The developer DAB was then added dropwise and incubated for 5 min. The digital images of each core were captured and stored using cameras of the ImageScope software interface.

The expression of FRα and FRβ was scored independently by two observers, and the observers did not know the clinical outcome data. The score took into account the intensity of staining and the positive rate of staining. The staining intensity was scored according to the staining depth of the cytoplasm or cell membrane in the sections. The staining scores were negative for no staining (0 points), weak for light yellow staining (1 point), medium for brown staining (2 points) and strong for yellowish brown staining (3 points). The positive staining rate was scored according to the cytoplasmic or cell membrane staining ratio in the sections. Three fields were selected for each locus, and a total of 50 cells were counted for each field. The average percentage of positive cells was counted and scored as follows: 0 points for ≤5%, 1 point for 6–25%, 2 points for 26–50%, 3 points for 51–75%, and 4 points for more than 76%. If the product of the above two scores was ≥6, the sample was labeled as high expression; otherwise, the sample was indicated to have low expression. The association between FRα/FRβ expression and overall survival was assessed by the Kaplan–Meier method, and statistical differences were determined by the log-rank test.

### Cell culture and in vitro transfection experiments

LL/2 cells, A549 cells, and MH-S cells were purchased from the American Type Culture Collection and cultured with folate-free Dulbecco’s modified Eagle’s medium (DMEM) supplemented with amikacin and 10% fetal bovine serum in incubators at 37 °C and 5% CO_2_.

In vitro transfection experiments were performed as described previously.^28^ F-PLP/eGFP containing 4 μg of pGFP was used to transfect LL/2 cells for 48 h in six-well plates. We used a FACS Calibur flow cytometer (BD Biosciences, USA) to determine the transfection efficiencies. Approximately 24 h after LL/2 and MH-S cell transfection with F-PLP/pBIM, PLP/pBIM, F-PLP/pVAX, or PLP/pVAX and glucose (5%), real-time quantitative RT-PCR was conducted to detect BIM gene expression. Approximately 48 h after the transfection of both cell lines, total protein was extracted for western blot analysis of BIM expression. Approximately 48 h after transfection, cells were harvested for flow cytometry analysis to detect necrotic cells.

### In vivo model of mouse lung cancer

Six- to eight-week-old female C57BL/6 mice and 4- to 5-week-old nude mice were purchased from HFK Bioscience (China), where they had been kept under specific pathogen-free (SPF) conditions. All mice were raised under SPF conditions in standard facilities at the State Key Laboratory of Sichuan University. All protocols were approved by the State Key Laboratory of Biotherapy Animal Care and Use Committee of Sichuan University in China. In vivo models were established by intravenous (i.v.) injection of LL/2 cells (~5 × 10^5^ cells/0.1 ml serum-free DMEM in C57BL/6 mice) and subcutaneous injection of A549 cells (~1 × 10^7^ cells/0.1 ml serum-free DMEM in nude mice). C57BL/6 mice were randomly allocated into five groups based on their body weights. Interventions were conducted 72 h after inoculation. Nude mice were randomly allocated into five groups based on their tumor volumes (~100 mm^3^). All mice were treated by i.v. injection once every 2 days with liposomes (30 μg) and plasmid DNA (5 μg) in 200 μl of 5% glucose. The mice were monitored daily for their living conditions, weighed every 2 days, and sacrificed when they appeared moribund. Then, whole blood, lungs, other vital organs, and tumor tissues were harvested, and the lung weight and the number of tumor nodules were recorded. Whole blood and blood serum were obtained for further use. Tumor tissues were divided into three sections: one was immediately embedded in optimum cutting temperature (OCT) compound for frozen sectioning, one was fixed with 4% paraformaldehyde in PBS (pH 7.4) and then embedded in paraffin for tissue sectioning, and the last was harvested for flow cytometry analysis. Other vital organs (the heart, liver, spleen, and kidney) were also harvested for analysis of tissue toxicity. The animal carcasses were disposed of by a professional institution (Dashuo Biotech, China).

### Real-time quantitative polymerase chain reaction (PCR)

Total RNA was isolated using an RNA Simple Total RNA Kit (TIANGEN, China) according to the manufacturer’s procedures and was quantified with a NanoDrop 2000 UV-Vis Spectrophotometer (Waltham, USA). Total RNA (1 μg per group) was reverse transcribed using a Prime Script^TM^ RT Reagent Kit with gDNA Eraser (TaKaRa, Japan). GAPDH was used as an internal control in this research. Primers were designed as follows: BIM-sense, 5′-CGCCGAATTCATGGCCAAGCAACCTTCTGA-3′; BIM-antisense, 5′-ACGCCTCGAGTCAATGCCTTCTCCATACCA-3′; GAPDH-sense, 5′-AACTTTGGCATTGTGGAAGG-3′; and GAPDH-antisense, 5′-ACACATTGGGGGTAGGAACA-3′. Quantitative real-time PCR was performed using a Bio-Rad CFX 96 with SsoAdvanced^TM^ Universal SYBR^®^ Green Supermix (Bio-Rad, USA). All experiments were performed in triplicate.

### Western blot analysis

Cells and tissues were lysed in RIPA buffer with protease inhibitor cocktail (Sigma-Aldrich, USA). Total protein concentrations were measured using a Bradford protein assay reagent kit (Bio-Rad, USA). Equal amounts of protein and a moderate-weight marker were loaded and separated by 12.5% SDS-PAGE before being transferred to Millipore polyvinylidene fluoride membranes. The membranes were blocked with 5% skim milk and incubated with primary antibodies for BIM (Cat# 2819, Cell Signaling Technology, USA) at 4 °C overnight. The membranes were visualized with a ChemiScope 5600 imaging system (Clinx Science, China) after incubation with a secondary antibody at 37 °C for 1 h. β-Actin (Santa Cruz, USA) was used as an internal control in this procedure.

### Flow cytometry analysis

LL/2 cells and MH-S cells were allowed to attach overnight and then treated with F-PLP/pBIM, PLP/pBIM, F-PLP/pVAX, or PLP/pVAX and 5% glucose injection. Flow cytometry analysis was carried out to detect apoptotic cells. After 48 h of incubation, the cells were labeled with PI and Annexin V (FITC Annexin V Apoptosis Detection Kit I, BD Biosciences, USA) according to the manufacturer’s instructions. LL/2 cells and MH-S cells, including the supernatant, were harvested, washed twice with PBS, and dispersed in 1× binding buffer. The cells were transferred into a staining solution with 100 µl of 1× binding buffer and incubated at room temperature for 15 min with 5 µl of PI and 5 µl of Annexin V before detection. Flow cytometry analysis was carried out on a FACS Calibur flow cytometer.

Ethical approval was obtained from the ethics committee of West China Hospital, Sichuan University. Fresh surgical specimens from lung cancer patients and tumor tissue specimens from mice were digested into single-cell suspensions and stained with CD45, F4/80, CD206, CD11c, FRβ, FRα, CD11b, Gr-1, CD19, CD3, CD4, CD8, CD44 or the corresponding isotypes. All antibodies were purchased from BD Pharmingen™ (USA) or BioLegend, Inc. (USA). Flow cytometry analysis was carried out on a FACS Calibur flow cytometer (BD Biosciences, USA), and the data were analyzed using NovoExpress software.

### Immunohistochemistry, immunofluorescence, and TUNEL assay

Immunohistochemical analysis for Ki67 was performed using paraffin sections. Tissues obtained from mice were fixed in 4% paraformaldehyde for 48 h and then embedded in paraffin for further use. The sections were deparaffinized in xylene twice for 10 min, rehydrated in an ethanol gradient for 2 min and rinsed in deionized water for 5 min. Then, the sections were treated with 10 mM citrate buffer (pH 6.0) for 3 min in an autoclave for antigen retrieval. A primary antibody for Ki67 was purchased from Abcam (Cat# ab16667, USA) and diluted 1:100 in PBS. The subsequent steps were similar to those of the immunohistochemistry analysis for FR described above.

Immunofluorescence analysis of microvessel density (MVD, CD31) was performed using frozen sections. Fresh tissues obtained from mice were embedded immediately in OCT compound. After they were sliced, the sections were fixed in cold acetone for 20 min, and endogenous peroxidase was inactivated by incubation with 3% H_2_O_2_ for 20 min at room temperature in the dark. Next, the sections were incubated with normal goat serum at 37 °C for 20 min to block nonspecific binding. A primary antibody for CD31 was purchased from Abcam (cat# ab28364, USA) and diluted 1:50 in PBS. The sections were incubated at 4 °C overnight with the primary antibody and PBS, and PBS was used as a control. A PE-labeled secondary antibody was supplied by Abcam. Negative control sections were exposed to the secondary antibody alone.

To detect apoptotic cells in tumor tissue, frozen sections were stained by terminal deoxynucleotidyl transferase-mediated dUTP nick end-labeling (TUNEL) with a DeadEndFluorometric TUNEL System kit (Promega, USA) in accordance with the manufacturer’s instructions.

The sections were observed and digitally photographed under a fluorescence microscope (Leica Microsystems, Germany). Quantification of MVD and Ki67-positive and TUNEL-positive cells was performed as described in a previous report.^33^ Five random fields at ×200 magnification were examined and counted for each section. All sections were observed by two investigators who were blinded.

Immunofluorescence analysis was performed on tumor sections to detect changes in macrophages after treatment in vivo with an F4/80 antibody (1:100, cat# 70076, Cell Signaling Technology, USA) and a cleaved-caspase 3 antibody (1:100, cat# 9662, Cell Signaling Technology, USA). After staining, the sections were observed under a ZEISS Imager Vario confocal microscope.

### Assessment of the safety and toxicity of F-PLP

To evaluate the toxicity of F-PLP in vivo, whole blood was used for routine blood examination using a fully automatic hematology analyzer (Nihon Kohden, Japan). Blood serum obtained through centrifugation was used for biochemical analysis with a fully automatic analyzer (Hitachi High-Technologies, Japan).^34^

### Statistical analysis

Statistical analysis was conducted using one-way ANOVA and Student’s *t* test in Statistical Product and Service Solutions software (SPSS V 19.0, IBM, USA). Differences with a *p* value < 0.05 were considered statistically significant. Survival was analyzed using Kaplan–Meier curves and log-rank tests.

## Results

### Baseline characteristics of lung cancer patients

The basic information of the 184 patients with pathologically confirmed lung cancer, 94 of which had adenocarcinoma, and 90 of which had squamous cell carcinoma, included in the tissue microarray analysis is shown in Table 1. The average age of the patients was 63 ± 9.4 years. The patients included 135 men and 49 women. Moderately differentiated cancer accounted for the majority of cases (59.2%). Approximately half of the patients were in AJCC stage III. Approximately half of the patients had lymph node metastases. One adenocarcinoma patient and one squamous cell carcinoma patient had distant metastases. Thirteen lung adenocarcinoma patients suffered EGFR gene mutations, and fifteen lung adenocarcinoma patients had ALK gene mutations. There were no genetic mutation statistics in the patients with lung squamous cell carcinoma.

**Table 1:** Baseline characteristics of patients included in the tissue microarray analysis.

	Adenocarcinoma (%)	Squamous cell carcinoma (%)	NSCLC (%)
Patients	94 (51.1)	90 (48.9)	184 (100)
Mean age (years/mean ± SD)	62.2 ± 9.9	63.8 ± 8.8	63 ± 9.4
Sex			
Male	51 (37.8)	84 (62.2)	135 (73.4)
Female	43 (87.8)	6 (12.2)	49 (26.6)
Differentiation			
III	11 (68.7)	5 (31.3)	16 (8.7)
II–III	18 (46.1)	21 (53.9)	39 (21.3)
II	57 (52.3)	52 (47.7)	109 (59.2)
I–II	5 (29.4)	12 (70.6)	17 (9.2)
I	3 (100)	0 (0)	3 (1.6)
AJCC stage			
I	22 (47.8)	24 (52.2)	46 (26.1)
II	21 (48.8)	22 (51.2)	43 (24.4)
III	46 (54.1)	39 (45.9)	85 (48.4)
IV	1 (50)	1 (50)	2 (1.1)
T stage			
T1	18 (54.6)	15 (45.4)	33 (18.3)
T2	43 (55.1)	35 (44.9)	78 (43.3)
T3	21 (43.7)	27 (56.3)	48 (26.7)
T4	12 (57.1)	9 (42.9)	21 (11.7)
N stage			
N0	39 (44.3)	49 (55.7)	88 (49.7)
N1	16 (55.2)	13 (44.8)	29 (16.4)
N2	16 (66.7)	8 (33.3)	24 (15.6)
N3	5 (100)	0 (0)	5 (2.8)
Nx	15 (48.4)	16 (51.6)	31 (17.5)
Metastasis			
Yes	1 (50)	1 (50)	2 (1.1)
No	93 (51.1)	89 (48.9)	182 (98.9)
EGFR			
Mutation	13	–	–
Wild type	81	–	–
ALK			
Mutation	15	–	–
Wild type	72	–	–

### High expression of FRα and FRβ in lung cancer cells and TAMs

**Fig. 1:**
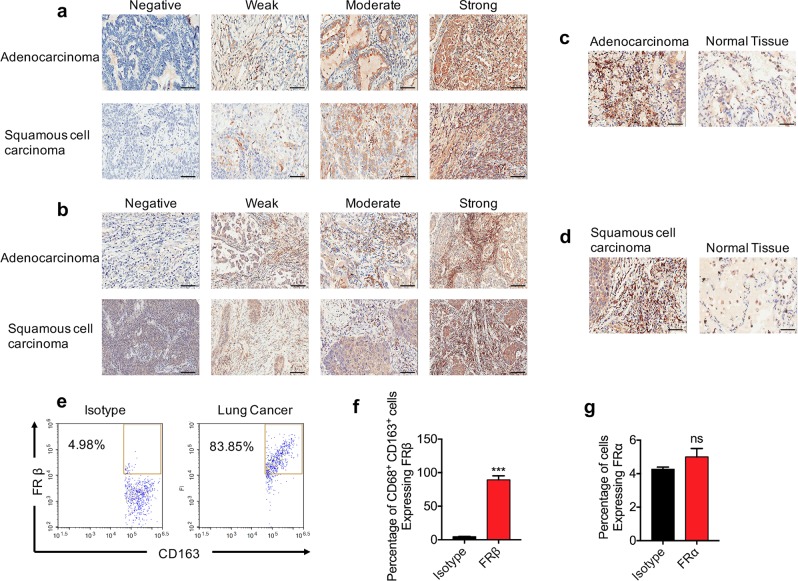
FRα and FRβ were overexpressed in human lung adenocarcinoma and squamous cell carcinoma, as determined by tissue microarray. **a** FRα was overexpressed in tumor cells of human lung adenocarcinoma and squamous cell carcinoma. **b** FRβ was highly expressed in the stromal tumor-associated macrophages of lung adenocarcinoma and squamous cell carcinoma. The expression of FRβ was higher in TAMs in lung adenocarcinoma (**c**) and squamous cell carcinoma (**d**) than in those in normal lung tissue. **e** CD68^+^ CD163^+^ M2 macrophages highly expressed FRβ in human lung cancer tissues. **f, g** Quantitative data from flow cytometry analysis of FRβ and FRα (magnification: ×200; scale bar: 50 μm; ***p < 0.001, ns: not significant).

The expression of FRα in lung cancer cells was analyzed. Typical pictures of no expression (0 points), weak expression (1 point), moderate expression (2 points), and strong expression (3 points) of FRα in both lung adenocarcinoma cells and lung squamous cell carcinoma cells are shown in Fig. 1a. The product of the staining intensity score and the staining positive rate score made a total score. A total score ≥6 was defined as high expression. We observed that FRα was highly expressed in both human lung adenocarcinoma cells and squamous cell carcinoma cells. A total of 89 patients with lung adenocarcinoma were included in the final analysis except for those with exfoliation. Among them, 53 patients had high expression of FRα, and the rate of high FRα expression in lung adenocarcinoma was 59.6%. A total of 87 patients with lung squamous cell carcinoma were included in the final analysis except for those with exfoliation. Among them, 29 patients had high expression of FRα, and the rate of high FRα expression in lung squamous cell carcinoma was 33.3%.

We also focused on the expression of FRβ in interstitial macrophages in non-small cell lung cancer. FRβ was overexpressed in interstitial macrophages in human lung adenocarcinoma and squamous cell carcinoma. Typical pictures of negative, weak, moderate, and strong expression are shown in Fig. 1b. A total of 92 patients with adenocarcinoma were included in the final analysis except for those with exfoliation. Among them, 49 patients had high expression of interstitial FRβ, and the rate of high interstitial FRβ expression in adenocarcinoma was 53.3%. A total of 89 patients with lung squamous cell carcinoma were included in the final analysis except for those with exfoliation. Among them, 42 patients had high interstitial FRβ expression, and the rate of high interstitial FRβ expression in lung squamous cell carcinoma was 47.2%. When compared with normal lung tissues, lung adenocarcinoma and lung squamous cell carcinoma tissues had higher FRβ staining intensity and a higher FRβ positive rate in macrophages (Fig. 1c, d).

To verify the expression of FRβ in M2 TAMs in lung cancer tissues, fresh lung cancer specimens were obtained and analyzed by flow cytometry. In the CD68^+^ CD163^+^ M2 macrophage population, FRβ expression in lung cancer tissues was ~84–96% (Fig. 1e, f). The expression of FRα in these lung cancer patients was negative (Fig. 1g).

### High FRβ expression is associated with poor cancer prognosis

**Fig. 2:**
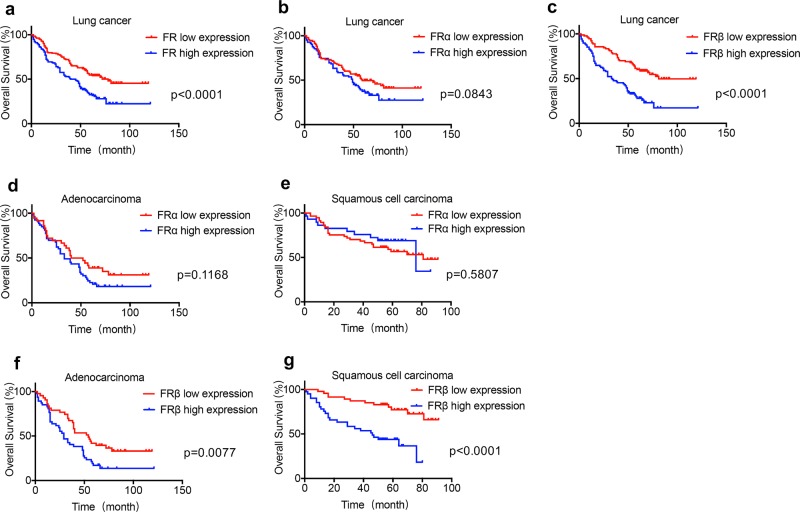
The overall survival between patients with high FR expression and low FR expression. **a** The difference in overall survival between lung cancer patients with high FR expression and low FR expression was significant (median OS: 42.5 vs. 78.0 months, HR = 0.5343, 95% CI 0.4054–0.7042, *p* < 0.0001). **b** The difference in overall survival between lung cancer patients with high FRα expression and low FRα expression was not significant (median OS: 49.0 vs. 59.0 months, HR = 0.7161, 95% CI 0.4849–1.058, *p* = 0.0843). **c** The overall survival between lung cancer patients with high FRβ expression and low FRβ expression was significant (median OS: 33.5 vs. 81.0 months, HR = 0.3972, 95% CI 0.2684–0.5876, *p* < 0.0001). The difference in overall survival between patients with high FRα expression and low FRα expression was not significant in lung adenocarcinoma (**d**) and squamous cell carcinoma (**e**) (lung adenocarcinoma: median OS: 33.0 vs. 46.0 months, HR = 0.6773, 95% CI 0.4186–1.096, *p* = 0.1168; squamous cell carcinoma: median OS: 76.0 vs. 81.0 months, HR = 1.224, 95% CI 0.6076–2.465, *p* = 0.5807). The difference in overall survival between patients with high FRβ expression and low FRβ expression was significant in lung adenocarcinoma (**f**) and squamous cell carcinoma (**g**) (lung adenocarcinoma: median OS: 29.0 vs. 54.0 months, HR = 0.5325, 95% CI 0.3296–0.8604, *p* = 0.0077; squamous cell carcinoma: median OS: 46.0 months vs. not reached, HR = 0.2672, 95% CI 0.1364–0.5234, *p* < 0.0001).

High expression of FR was associated with poor survival outcome in lung cancer patients (Fig. 2a). The median survival time of patients with high FR expression was 42.5 months, whereas the median survival time of patients with low FR expression was 78 months. The difference was statistically significant (*p* < 0.0001, HR = 0.5343, 95% CI 0.4054–0.7042).

To investigate whether the FR could be used as an independent prognostic predictor in NSCLC, as shown in Table 2, we converted the risk factors associated with lung cancer prognosis into two-category variables for stratified analysis, including sex (male/female), age (<60 years old/≥60 years old), pathological grade (I–II grades/III grade), tumor size (≤5 cm/>5 cm), T stage (T1-T2/T3-T4), N stage (N0/N1-N3), AJCC stage (I–II/III–IV), adenocarcinoma EGFR gene status (wild type/mutant type), and ALK gene status (wild type/mutant type). We found that AJCC stage and N stage were significantly associated with overall survival (*p* < 0.05). Combined with clinical experience, all the above risk factors were included in multivariate regression analysis. We found that the FR was an independent prognostic factor in NSCLC (*p* = 0.000, 95% CI 1.319–2.531).

**Table 2:** Stratified analysis of risk factors associated with overall survival in lung cancer.

	Adenocarcinoma		Squamous cell carcinoma		NSCLC	
	*P* value	HR (95%CI)	*P* value	HR (95%CI)	*P* value	HR (95%CI)
Sex (male/female)	0.1905	1.362 (0.8522–2.177)	0.746	0.8248 (0.2292–2.968)	0.3378	0.8214 (0.5381–1.254)
Age (<60/≥60 years)	0.8353	1.052 (0.648–1.707)	1.1409	0.5748 (0.2936–1.125)	0.4815	0.8669 (0.5854–1.284)
Differentiation (I–II/III)	0.8882	0.9645 (0.5763–1.614)	0.7462	0.8913 (0.4342–1.83)	0.6634	0.913 (0.5992–1.391)
Tumor size (≤5 cm/>5 cm)	0.0249	0.5321 (0.2631–1.076)	0.7972	0.914 (0.454–1.84)	0.6837	0.9147 (0.5875–1.424)
T stage (T1–T2/T3–T4)	0.0225	0.5836 (0.3464–0.9833)	0.9508	0.9789 (0.4938–1.941)	0.2432	0.7943 (0.5308–1.188)
N stage (N0/N1–N3)	0.0002	0.3882 (0.2244–0.6716)	0.0081	0.4001 (0.172–0.9307)	<0.0001	0.3668 (0.231–0.5827)
AJCC stage (I–II/III–IV)	<0.0001	0.3265 (0.1831–0.5823)	0.008	0.393 (0.1728–0.8935)	<0.0001	0.3437 (0.2139–0.5523)
EGFR (wild type/mutation)	0.0578	0.5663 (0.2708–1.184)	–	–	–	–
ALK (wild type/mutation)	0.3511	1.37 (0.7457–2.518)	–	–	–	–

When distinguishing FR subtypes, lung cancer patients with high FRβ expression had a poor prognosis. The median survival time of lung cancer patients with high FRα expression was 49 months, while the median survival time of lung cancer patients with low FRα expression was 59 months. The statistical difference between patients with high FRα expression and low FRα expression was not significant (*p* = 0.0843, HR = 0.7161, 95% CI 0.4849–1.058) (Fig. 2b). However, the difference between patients with FRβ high expression and low expression was statistically significant (*p* < 0.0001, HR = 0.3972, 95% CI 0.2684–0.5876). The median survival time of lung cancer patients with high FRβ expression was 33.5 months, and the median survival time of patients with low FRβ expression was 81 months (Fig. 2c).

To determine the predictive effect of FRα and FRβ on the prognosis of NSCLC, we also performed multivariate regression analysis. All risk factors were included in the analysis. We found that FRα could not be used as a predictor of lung cancer prognosis (*p* = 0.356, 95% CI 0.788–1.938), whereas FRβ could be used as an independent predictor of lung cancer prognosis (*p* = 0.000, 95% CI 1.794–4.913).

The relationship between FRα expression and the prognosis of lung adenocarcinoma and lung squamous cell carcinoma was analyzed. As shown in Fig. 2d, in lung adenocarcinoma, there was no significant difference in the overall survival between patients with high FRα expression and those with low FRα expression (*p* = 0.1168, HR = 0.6773, 95% CI 0.4186–1.096). The median survival of patients with high FRα expression was 33 months. The median survival of patients with low FRα expression was 46 months. As shown in Fig. 2e, in lung squamous cell carcinoma, there was no significant difference in the overall survival between patients with high FRα expression and those with low FRα expression (*p* = 0.580, HR = 1.224, 95% CI 0.6076–2.4657). The median survival time of patients with high FRα expression in lung squamous cell carcinoma was 76 months, and the median survival of patients with low FRα expression was 81 months. There was no statistically significant association between FRα expression and overall survival in lung adenocarcinoma and squamous cell carcinoma.

Further multivariate regression analysis showed that the risk factors associated with the prognosis of lung adenocarcinoma were T stage, N stage, and AJCC stage. The risk factors associated with the prognosis of lung squamous cell carcinoma were N stage and AJCC stage (Table 2). Combined with clinical experience, all risk factors were included in the regression analysis. We found that high expression of FRα had no predictive value for the prognosis of lung adenocarcinoma and lung squamous cell carcinoma (*p* = 0.435 and *p* = 0.426, respectively).

The relationship between the expression of FRβ in interstitial TAMs and the prognosis of lung adenocarcinoma and lung squamous cell carcinoma was further analyzed. As shown in Fig. 2f, a significant difference existed in the overall survival between patients with high FRβ expression and those with low FRβ expression in interstitial TAMs in lung adenocarcinoma (*p* = 0.0077, HR = 0.5325, 95% CI 0.3296–0.8604). The median survival time of lung adenocarcinoma patients with high FRβ expression was 29 months, and the median survival time of patients with low FRβ expression was 54 months. As shown in Fig. 2g, in lung squamous cell carcinoma, the difference in overall survival between patients with high FRβ expression in stromal TAMs and patients with low FRβ expression was statistically significant (*p* < 0.0001, HR = 0.2672, 95% CI 0.1364–0.5234). The median survival time of lung squamous cell carcinoma patients with high FRβ expression was 46 months, and the median survival time of patients with low FRβ expression was not reached.

Further multivariate regression analysis was performed. All risk factors were included in the regression analysis. We found that high FRβ expression was an independent predictor of lung adenocarcinoma prognosis (*p* = 0.003, 95% CI 1.491–6.536) and an independent predictor of lung squamous cell carcinoma prognosis (*p* = 0.002, 95% CI 1.793–12.465).

### Preparation and characterization of F-PLP and F-PLP/pBIM

**Fig. 3:**
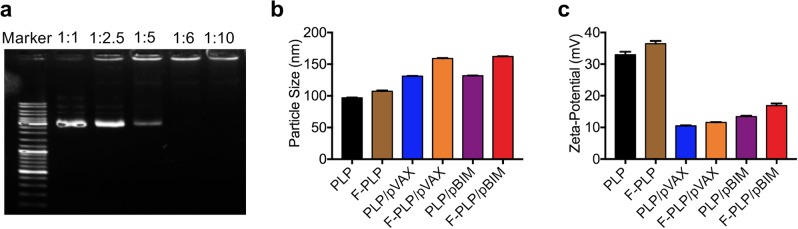
Physicochemical properties of F-PLP/pBIM. **a** Gel retardation assay of DNA and lipoplexes. Lane 1, DNA marker; lanes 2–6, mixed pBIM with F-PLP at 1:1, 1:2.5, 1:5, 1:6, and 1:10 ratios. The BIM plasmid was completely incorporated into F-PLP, and lipoplexes were prepared without free DNA when pBIM was mixed with F-PLP at a 1:6 ratio. **b** Particle size of liposomes and lipoplexes. **c** Zeta potential of liposomes and lipoplexes.

F-PLP was produced by utilizing membrane hydration methods described previously.^26,27^ The optimal binding ratio of F-PLP and pBIM was determined by PCR. As shown in Fig. 3, when the mass ratio of folate liposome to plasmid was 6:1, the folate liposome could completely encapsulate the plasmid. The particle sizes of PLP and F-PLP were ~97 and 107 nm, respectively. The liposome/plasmid complexes had particle sizes of 130–160 nm, which were significantly larger than those of the corresponding individual liposomes. The zeta potential of F-PLP and PLP was ~35 mV. The zeta potential of the liposome/plasmid complex was 13–17 mV, which was significantly lower than that of the liposome alone.

### Potential toxicity of F-PLP and LP

**Fig. 4:**
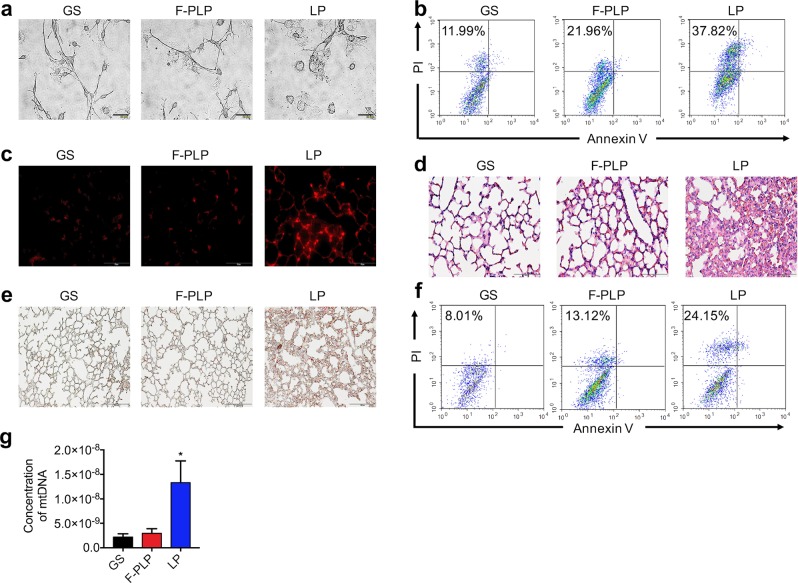
The safety and toxicity assessment of F-PLP in mice. **a** Morphological changes in primary lung epithelial cells treated with F-PLP (50 μg/ml) and LP (50 μg/ml) in vitro for 30 min. Cells were subjected to inverted microscopy analysis. **b** A representative image of the necrotic cells induced by F-PLP (50 μg/ml) and LP (50 μg/ml) in vitro for 30 min as detected by flow cytometry with Annexin V and PI staining. **c** Propidium iodide (PI)-positive necrotic cells in mouse lungs. F-PLP and LP were injected through the tail vein of mice, and 2 h later, PI and 4% formaldehyde were perfused through the tail vein for the detection of necrotic cells. **d, e** Pulmonary inflammation induced by F-PLP and LP upon systemic injection in mice. C57BL/6 mice were injected with F-PLP (25 mg/kg) and LP (25 mg/kg). HE staining (**d**) and specific esterase staining of neutrophils (**e**) in representative mouse lung sections 24 h after injection are presented. **f** A representative image of the necrotic cells induced by the injection of F-PLP and LP in vivo as detected by flow cytometry with Annexin V and PI staining. C57BL/6 mice were injected with F-PLP or LP liposomes (25 mg/kg). Necrotic cells in BAL fluid were detected 4 h after injection by flow cytometry with Annexin V and PI staining. **g** Primary lung epithelial cells were treated with F-PLP (50 μg/ml) and LP (50 μg/ml) for 4 h, and the supernatant was collected and concentrated for mtDNA determination (*p < 0.05 compared with the control group).

To examine the potential toxicity of F-PLP, we used a common cationic liposome gene delivery system as a positive control. We collected primary lung epithelial cells to compare the toxicity of F-PLP and LP in vitro. Microscopic observation demonstrated that LP rapidly induced primary lung epithelial cell swelling and fracture, whereas F-PLP did not (Fig. 4a). Moreover, there were many fewer necrotic cells in the F-PLP group than in the LP group in vitro and in vivo, as shown in Fig. 4b, c. Much less pulmonary inflammation was induced by F-PLP than by LP, as shown in Fig. 4d, e. Bronchoalveolar lavage fluid was examined for cell necrosis induced by the injection of F-PLP and LP in vivo by flow cytometry with Annexin V and PI staining. There were many more necrotic cells in the LP group than in the F-PLP group, as shown in Fig. 4f. Our previous study demonstrated that cationic liposome-induced necrotic cells could result in mtDNA release and cause subsequent inflammatory responses.^34^ Primary lung epithelial cells were treated with F-PLP (50 μg/ml) or LP (50 μg/ml) for 4 h, and the supernatant was collected and concentrated for mtDNA determination. The release of mtDNA in the LP group was much greater than that in the F-PLP group, as shown in Fig. 4g.

### In vitro F-PLP-mediated gene expression and biological activity of pBIM in the LL/2 cell line

**Fig. 5:**
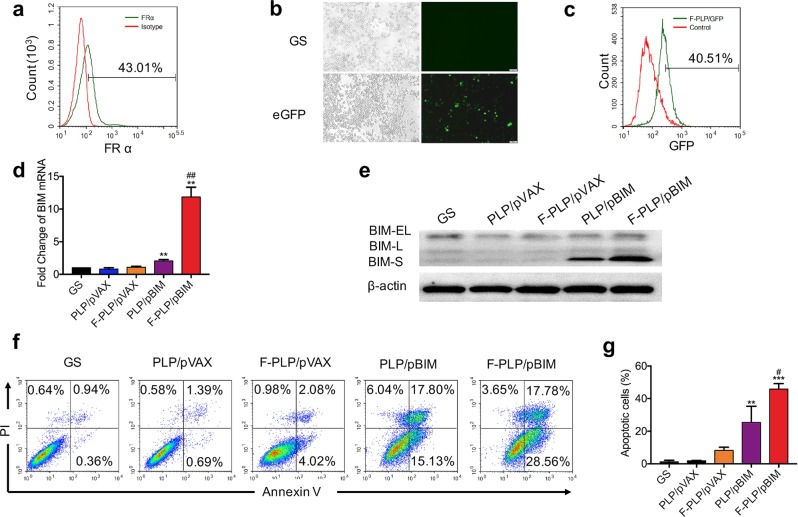
In vitro transfection activity of F-PLP and antitumor effect of F-PLP/pBIM. **a** The FRα expression rate of the LL/2 cell line was 43.01%. **b, c** In vitro transfection activity of F-PLP/eGFP evaluated by microscopic and flow cytometry analyses. **d, e** qRT-PCR and western blot analysis of BIM expression at the mRNA level and protein level in each group. BIM expression was significantly higher in the F-PLP/pBIM group than in the other groups. **f** Flow cytometry analysis of PI and Annexin V staining after in vitro transfection. F-PLP/pBIM transfection was able to increase the quantity of apoptotic cells at both early and later stages. **g** The statistical data of apoptotic LL/2 cells from the flow cytometry analysis (magnification: ×200; scale bar: 50 μm; **p < 0.01 and ***p < 0.001 vs. control, ^#^*p* < 0.05 and ^##^*p* < 0.01 vs. PLP/pBIM).

The LL/2 cell line overexpressed FRα. The expression rate was ~43%, as shown in Fig. 5a. The transfection efficiency of F-PLP was ~40% in LL/2 cells, as evaluated by flow cytometry analysis and microscopic observation, which demonstrated that F-PLP could condense plasmid DNA and be transfected into LL/2 cells effectively through interactions between folate and FRs (Fig. 5b, c). qRT-PCR and western blot analysis demonstrated that BIM-S mRNA and protein were expressed at higher levels in the cells of the F-PLP/pBIM group than in the cells of the other groups (Fig. 5d, e). Moreover, apoptosis analysis showed that F-PLP/pBIM transfection was able to increase the quantity of apoptotic cells at both the early and late stages, as shown in Fig. 5f. The statistical data regarding apoptotic cells are shown in Fig. 5g. In summary, F-PLP was successfully transfected into LL/2 cells in vitro, and F-PLP/pBIM contributed to the enhanced biological activity of BIM-S.

### In vitro F-PLP-mediated gene expression and biological activity of pBIM in macrophages

**Fig. 6:**
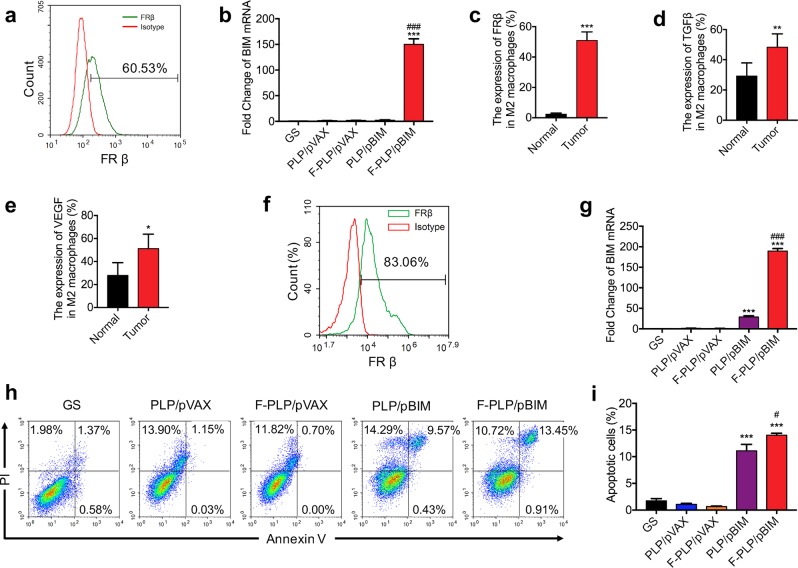
In vitro F-PLP-mediated gene expression and biological activity of the BIM plasmid in macrophages. **a** The FRβ expression rate of MH-S macrophages. **b** qRT-PCR analysis of BIM expression at the mRNA level in each group. BIM expression in the F-PLP/pBIM group was significantly higher than that in the control group. **c** Flow cytometry analysis demonstrated that the expression of FRβ in CD45^+^ F4/80^+^ CD206^+^ M2 macrophages was significantly higher in lung cancer metastatic tissues than in normal lung tissues. **d, e** The expression of TGFβ and VEGF in CD45^+^ F4/80^+^ CD206^+^ M2 macrophages was significantly higher in lung cancer metastatic tissues than in normal lung tissues. **f** FRβ expression rate of bronchoalveolar lavage macrophages. **g** qRT-PCR of BIM expression in each group. BIM expression in the F-PLP/pBIM-treated group was significantly higher than that in the other groups at the mRNA level. **h** Flow cytometry analysis of PI and Annexin V staining in each group. The quantity of apoptotic cells in F-PLP/pBIM-treated MH-S cells was significantly increased. **i** The statistical data of apoptotic MH-S cells from the flow cytometry analysis (***p < 0.001 vs. control; ^#^*p* < 0.05 and ^###^*p* < 0.001 vs. PLP/pBIM).

To study the F-PLP-mediated antitumor activity of the BIM plasmid in macrophages, we selected the MH-S macrophage cell line. The MH-S cell line overexpressed FRβ. The expression rate was ~60%, as shown in Fig. 6a. F-PLP could condense plasmid DNA and be transfected into MH-S cells effectively through interactions between folate and FRs. qRT-PCR analysis demonstrated that BIM-S mRNA was expressed at higher levels in the F-PLP/pBIM group than in the other groups (Fig. 6b).

Moreover, we demonstrated that the expression of FRβ in M2 macrophages was significantly higher in lung cancer metastatic tissues than in normal lung tissues (Fig. 6c). The intracellular cytokines produced by M2 macrophages, such as TGFβ and VEGF, were increased in lung cancer metastatic tissues compared with normal lung tissues (Fig. 6d, e). Then, we established a primary macrophage model in vitro with bronchoalveolar lavage macrophages. The FRβ expression rate in bronchoalveolar lavage macrophages was ~83.06%, as shown in Fig. 6f. BIM-S expression in the F-PLP/pBIM-treated group was significantly higher than that in the other groups at the mRNA level (Fig. 6g).

Furthermore, the apoptotic analysis showed that F-PLP/pBIM transfection was able to increase the quantity of apoptotic cells at both the early and late stages, as shown in Fig. 6h. The statistical data regarding apoptotic cells are shown in Fig. 6i.

### In vivo antitumor activity in tumor tissues

**Fig. 7:**
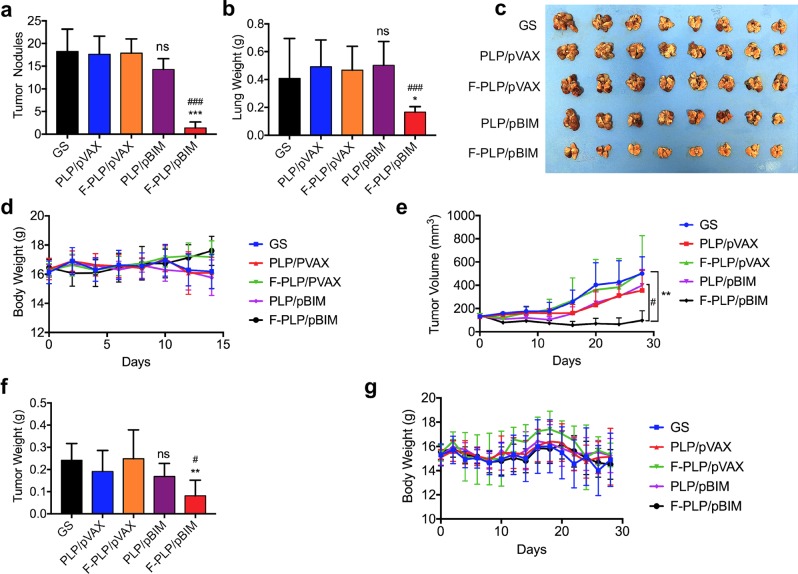
In vivo antitumor activity of F-PLP/pBIM in FRα-positive and FRα-negative lung cancer models. **a–d** In vivo antitumor activity in the FRα-positive LL/2 lung cancer model. **a** Number of tumor nodules in different groups (GS, PLP/pVAX, F-PLP/pVAX, PLP/pBIM, and F-PLP/pBIM-treated mice). **b** Lung weight in different groups. **c** Images of lungs and corresponding tumors. **d** Body weight. **e–g** In vivo antitumor activity in the FRα-negative A549 lung cancer model. **e** Tumor volume in different groups (GS, PLP/pVAX, F-PLP/pVAX, PLP/pBIM, and F-PLP/pBIM-treated mice). **f** Tumor weight in different groups. **g** Body weight. (**p < 0.01 and ***p < 0.001 vs. control; ns: not significant vs. control; ^#^*p* < 0.05 vs. PLP/pBIM).

First, we selected the FRα-positive LL/2 mouse lung cancer model. As shown in Fig. 7a–d, i.v. administration of F-PLP/pBIM resulted in less tumor growth, as represented by both the weight of the lungs and the total number of tumor nodules, than that seen in the control treatment. A significant difference was observed between the PLP/pBIM-treated group and the F-PLP/pBIM-treated group. This indicated that the F-PLP/pBIM therapy showed more effectiveness than the PLP/pBIM therapy.

To evaluate the efficiency of F-PLP/pBIM in an FRα-negative mouse model, we chose the A549 subcutaneously implanted lung cancer model in nude mice. As shown in Fig. 7e–g, the tumor weight of the F-PLP/pBIM treatment group was significantly less than that of the control group and the PLP/pBIM group. The growth of tumors in the F-PLP/pBIM-treated group was significantly slower than that in the other groups.

### Effects on angiogenesis, proliferation and apoptosis

F-PLP/pBIM showed a considerable antitumor effect by vector targeting, although the mechanisms need further study. We investigated changes in angiogenesis (CD31), cell proliferation (Ki67), and cell apoptosis (TUNEL) in lung cancer cells in the LL/2 mouse lung cancer model to explore the antitumor mechanisms of F-PLP/pBIM.

**Fig. 8:**
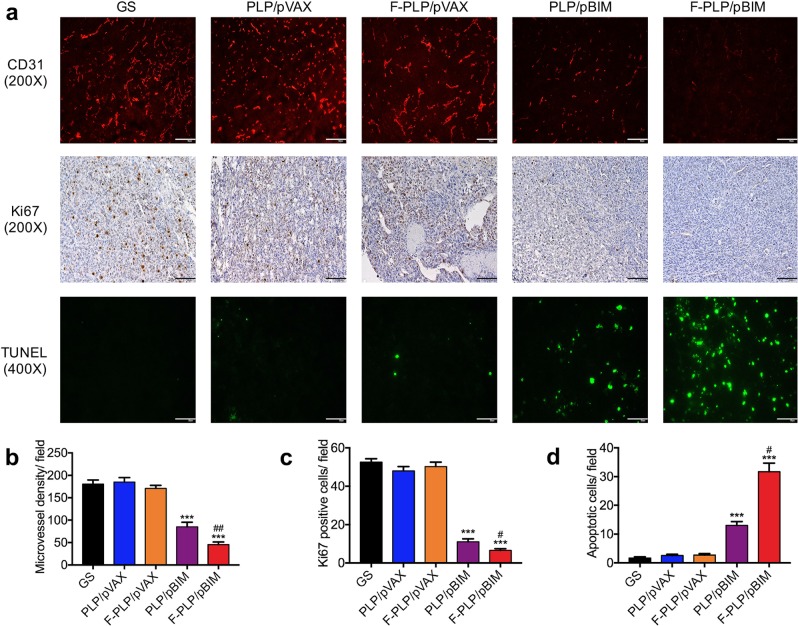
The effect of F-PLP/pBIM on angiogenesis, proliferation, and apoptosis in vivo. **a** The immunofluorescence and IHC results of CD31, Ki67, and TUNEL staining. Representative sections of each group are presented. **b–d** The number of CD31-positive vessels, Ki67-positive cells, and TUNEL-positive cells in the field (five high-power fields per slide, ***p < 0.001 vs. control; ^#^*p* < 0.05 and ^##^*p* < 0.01 vs. PLP/pBIM).

First, to investigate the effect of F-PLP/pBIM on angiogenesis, CD31 expression was examined by immunofluorescence staining. As shown in Fig. 8a, angiogenesis in the F-PLP/pBIM group was significantly less than that in the other groups. F-PLP/pBIM sharply reduced the number of CD31-positive vessels and reduced MVD to levels significantly lower than those of the PLP/pBIM (*p* < 0.01) and control (*p* < 0.001) groups, as shown in Fig. 8b.

Ki67 immunohistochemical staining was used to evaluate tumor cell proliferation. As shown in Fig. 8a, tumor cell proliferation in the F-PLP/pBIM group was significantly lower than that in the other groups. There were many fewer Ki67-positive cells upon microscopic observation in the F-PLP/pBIM group than in the PLP/pBIM (*p* < 0.05) and control (*p* < 0.001) groups, as shown in Fig. 8c.

Then, the TUNEL method was utilized to investigate tumor cell apoptosis. TUNEL-positive cells were counted, and as shown in Fig. 8a, many strongly positive nuclei identified as apoptotic could be observed in tumor tissue treated with F-PLP/pBIM, but such nuclei were rarer in the PLP/pBIM group and in the other groups. As shown in Fig. 8d, tumor cell apoptosis in the F-PLP/pBIM group was much higher than that in the PLP/pBIM (*p* < 0.05) and control (*p* < 0.001) groups.

### F-PLP/pBIM, M2 macrophages, and the tumor microenvironment in vivo

**Fig. 9:**
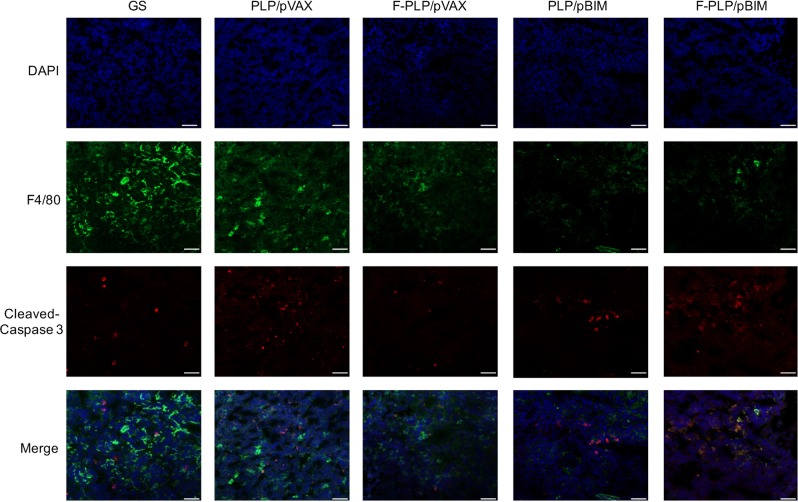
Confocal micrographs of tumor tissues with F4/80 and cleaved caspase 3 immunofluorescent staining. The apoptosis of macrophages in the F-PLP/pBIM group was significantly increased compared with that in the other groups. Representative sections from each group are presented (magnification: ×200; scale bar: 50 μm).

Confocal micrographs of tumor tissues in the LL/2 mouse lung cancer model with F4/80 and cleaved caspase 3 immunofluorescent staining are shown in Fig. 9. We found that the apoptosis of macrophages in the F-PLP/pBIM-treated group was significantly higher than that of macrophages in the other groups.

**Fig. 10:**
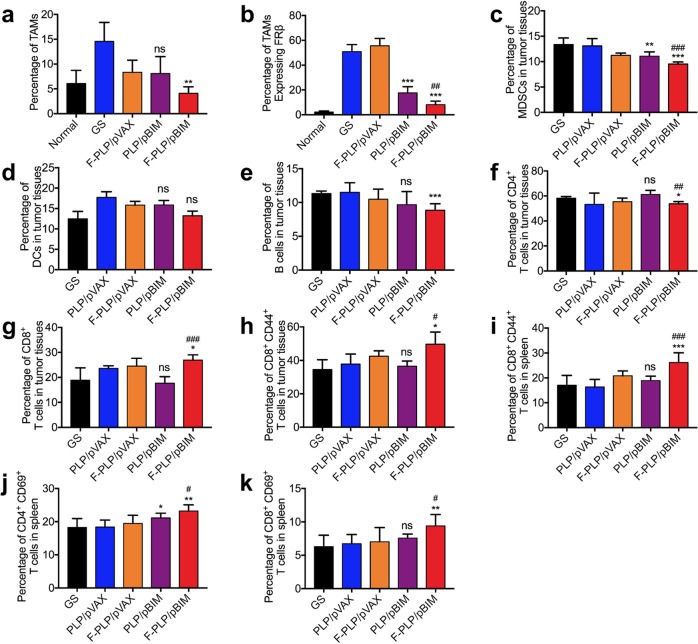
Changes in the tumor microenvironment after F-PLP/pBIM treatment in vivo. Quantitative data from flow cytometry analysis of CD45^+^ F4/80^+^ CD206^+^ M2 macrophages (**a**), CD45^+^ F4/80^+^ CD206^+^ FRβ^+^ TAMs (**b**), CD45^+^ CD11b^+^ Gr-1^+^ MDSCs (**c**), CD45^+^ CD11b^+^ CD11c^+^ DCs (**d**), CD45^+^ CD11b^+^ CD19^+^ B cells (**e**), CD3^+^ CD4^+^ T cells (**f**), CD3^+^ CD8^+^ T cells (**g**), and CD3^+^ CD8^+^ CD44^+^ memory T cells (**h**) in tumor tissues and CD3^+^ CD8^+^ CD44^+^ memory T cells (**i**), CD3^+^ CD4^+^ CD69^+^ activated T cells (**j**), and CD3^+^ CD8^+^ CD69^+^ activated T cells (**k**) in spleens (ns: not significant, *p < 0.05, **p < 0.01 and ***p < 0.001 vs. control; ^#^*p* < 0.05, ^##^*p* < 0.01, and ^###^*p* < 0.001 vs. PLP/pBIM).

We found that the number of M2 macrophages (CD45^+^ F4/80^+^ CD206^+^) was significantly increased after LL/2 cell injection but was decreased in vivo after F-PLP/pBIM treatment compared with that in the control group (Fig. 10). In addition, the number of FRβ-positive macrophages in the F-PLP/pBIM group was significantly reduced compared with that in the control group and the PLP/pBIM group, demonstrating that the F-PLP/pBIM increases the therapeutic effect.

The number of MDSCs in the tumor microenvironment of the F-PLP/pBIM treatment group were significantly reduced compared with that in the control group and the PLP/pBIM group. The differences were statistically significant. DCs were not significantly decreased in the F-PLP/pBIM treatment group. B cell numbers in the F-PLP/pBIM treatment group were significantly reduced compared with those in the control group, while PLP/pBIM treatment had no significant effect on B cells.

CD3^+^ CD4^+^ T cell numbers in the F-PLP/pBIM treatment group were significantly reduced compared with those in the control group, while the CD3^+^ CD8^+^ T cell numbers in the F-PLP/pBIM group were significantly increased compared with those in the control group and the PLP/pBIM group. The CD3^+^ CD8^+^ CD44^+^ memory T cell numbers in the F-PLP/pBIM group were significantly increased compared with those in the control group and the PLP/pBIM group. The CD3^+^ CD8^+^ CD44^+^ memory T cell numbers, CD4^+^ CD69^+^ activated T cell numbers and CD8^+^ CD69^+^ activated T cell numbers in spleens in the F-PLP/pBIM group were significantly increased compared with the respective numbers in the control group and the PLP/pBIM group.

### Assessment of safety and toxicity of F-PLP in mice

To preliminarily investigate the safety of F-PLP in vivo, we observed the appearance, body weight, and fecal and urinary excretions of the mice, and no toxicity was found. We conducted routine blood examinations and blood biochemistry analyses. No significant differences in alanine aminotransferase, aspartate aminotransferase, creatinine, urea nitrogen, hemoglobin concentration or platelet quantity were observed among the groups. All these results are shown in Supplementary Figs. 1 and 2.

## Discussion

Stromal TAMs are associated with poor prognosis in non-small cell lung cancer.^35,36^ Some studies seek to find reliable targets in TAMs, but currently, such targets are rare.^37^ The FR, a high-affinity membrane folate-binding protein, has been found to be overexpressed in various tumors.^38–43^ The efficacy of folate liposomal complexes in tumor-specific targeted therapies has been demonstrated.^28,32,44^ However, most research is limited to tumor types with high FRα expression in tumor cells. We found that FRβ was highly expressed in M2 macrophages and may be an ideal target in lung cancer. However, there is a lack of corresponding treatments to target M2 macrophages with FR-related therapeutics. Therefore, we demonstrated the feasibility of using FRβ as a therapeutic target for TAMs in the microenvironment of lung cancer and developed a folate-modified lipoplex comprising a F-PLP delivering a pBIM to target lung cancer. Studies have shown that ordinary cationic liposomes have significant cytotoxicity.^34^ The F-PLP used in our study blocks some of the positive charges because of the folate modification, so the toxicity is greatly reduced. Hence, targeting tumor cells and/or TAMs by the folate liposomal complex (F-PLP/pBIM) can achieve considerable therapeutic effects with tolerable toxicity.

In this study, we confirmed that FRα and FRβ were highly expressed in tumor cells and stromal TAMs, respectively, by microarray analysis of lung adenocarcinoma and lung squamous cell carcinoma. Moreover, high expression of interstitial FRβ was associated with poor prognosis in lung adenocarcinoma and lung squamous cell carcinoma. FRβ could be used as an independent predictor of lung squamous cell carcinoma and lung adenocarcinoma prognosis. Based on this, we designed and produced a F-PLP using the membrane dispersion method, which is less toxic than common cationic liposomes and could be used as a gene carrier to specifically bind the pBIM. Delivery to lung cancer cells and TAMs with F-PLP/pBIM in vitro and in vivo achieved considerable therapeutic effects. TAMs in the tumor microenvironment were significantly depleted with no significant organ or hematological toxicity.

The rates of high FRα expression in lung adenocarcinoma and lung squamous cell carcinoma were 59.6% and 33.3%, respectively. The rates of high stromal FRβ expression in lung adenocarcinoma and lung squamous cell carcinoma were 53.3% and 47.2%, respectively. The median survival time of patients with high FR expression was 42.5 months, whereas it was 78 months for patients with low FR expression. There was no significant difference in the overall survival between patients with high and low FRα expression. However, the prognosis of patients with high FRβ expression was much worse than that of patients with low FRβ expression. The median survival time of patients with high FRβ expression was 33.5 months, while that of patients with low FRβ expression was 81 months. The differences were statistically significant. Multivariate regression analysis suggested that FRβ could be used as an independent predictor of lung adenocarcinoma and lung squamous cell carcinoma. This indicated the prognostic role of FRβ in TAMs, which could serve as a potential target for lung cancer treatment.

We mixed F-PLP with the pBIM through electrostatic interactions. It was shown by agarose gel electrophoresis that the pBIM was completely incorporated into F-PLP when the ratio of pBIM to F-PLP was 1:6. The primary properties of liposomes and lipoplexes were characterized. The positive charge of liposomes was neutralized when the negatively charged pBIMs were bound. As a result, the zeta potential values of all lipoplexes were much lower than those of the liposomes. The sizes of the liposomes were significantly increased by combination with the pBIMs.

In this study, a powerful antitumor effect of F-PLP/pBIM was demonstrated. F-PLP/eGFP was transfected into FRα-positive LL/2 cells and FRβ-positive macrophages effectively through interactions between folate and the FR. The transfection efficiency was ~40%. The expression of BIM-S protein was significantly increased, resulting in the apoptosis of tumor cells and macrophages.

In vivo antitumor experiments showed that F-PLP/pBIM significantly inhibited lung cancer growth. In the FRα-positive LL/2 model, the number of tumor nodules and the lung weight were significantly lower in the F-PLP/pBIM-treated mice than in the control and PLP/pBIM-treated mice. Here, we demonstrated that additional FR targeting contributed to a significant difference in antitumor activity between F-PLP/pBIM and PLP/pBIM. This difference in antitumor effect could be attributed to the specific interactions and effective uptake of folate in F-PLP/pBIM and FR-expressing tumor cells and TAMs. To confirm that the F-PLP/pBIM complex could specifically target TAMs, we selected the FRα-negative A549 model.^45^ We found that F-PLP/pBIM also significantly inhibited the growth of subcutaneous tumors. However, the nontargeted PLP/pBIM treatment group had no significant therapeutic effect. The F-PLP/pBIM complex could specifically target TAMs and achieved considerable effects in treating lung cancer.

The potential mechanisms of F-PLP/pBIM-based therapy were examined by CD31 staining, Ki67 staining, and TUNEL assays. F-PLP/pBIM sharply reduced MVD, significantly inhibited tumor cell proliferation, and significantly promoted tumor cell apoptosis. These mechanisms might have contributed to the substantial antitumor effect of F-PLP/pBIM. Pathological angiogenesis plays a crucial role in tumor growth, dissemination, and metastasis.^46^ Reports have also indicated that BIM has antiangiogenic activity.^47^ As reported in our study, F-PLP/pBIM suppressed tumor-associated angiogenesis more effectively than the other treatments. BIM has been shown in previous studies to have an impact on tumor cell proliferation in non-small cell lung cancer.^48^ This finding is supported by our current data indicating that F-PLP/pBIM significantly suppresses tumor proliferation, as determined by Ki67 expression analysis. BIM-S has been reported to be the most potent isoform in inducing apoptosis.^49^ We also demonstrated that the induction of apoptosis in the F-PLP/pBIM group was much greater than that in other groups by TUNEL staining, and the percentage of apoptosis of folate-targeted cells was greater than that of nontargeted cells.

Upon treating LL/2-bearing mice with F-PLP/pBIM, we also examined apoptosis of TAMs and changes in the tumor microenvironment. It has been reported that apoptosis in macrophages is potentially mediated by the activation and upregulation of BIM.^50^ FRβ is highly expressed in TMAs.^19^ In our work, we used immunofluorescence methods to detect the expression of F4/80 and cleaved-caspase 3. Apoptosis of macrophages in the F-PLP/pBIM group was significantly higher than that in the other groups. TAMs are similar to M2 macrophages phenotypically and functionally.^51,52^ We found that the number of TAMs significantly increased after tumor cell injection as the expression of FRβ increased. However, the number of TAMs was significantly lower in the F-PLP/pBIM group than in the other groups. In addition, FRβ expression was reduced. The tumor microenvironment was altered. Compared with the control group, the immune cells in the tumor microenvironment that promote tumor progression, such as MDSCs,^53^ B cells^54^, and CD4^+^ T cells,^55^ were significantly reduced, whereas the immune cells that have antitumor roles, such as CD8^+^ T cells^56^ and CD44^+^ memory T cells,^57^ were significantly increased. Therefore, BIM-S and FRα/β targeting using F-PLP/pBIM exhibited synergistic antitumor effects in lung cancer treatment. Gene targeting mediated by F-PLP/pBIM with a nonviral vector could significantly inhibit lung cancer growth and had optimal antitumor efficiency.

Finally, the safety and toxicity of F-PLP/pBIM were preliminarily evaluated in mice. No obvious toxicity was observed, as determined by appearance, body weight, and fecal and urinary excretion. The morphology of vital organs as examined by H&E staining was normal. No ischemia or necrosis was observed. Routine blood tests and blood biochemistry analyses found no differences in liver and kidney function, myocardial enzymes, blood glucose, blood lipids, or other indicators.

## Conclusions

FRα and FRβ are highly expressed in non-small cell lung cancer tumor cells and stromal TMAs, respectively. High interstitial FRβ expression is associated with poor prognosis in lung adenocarcinoma and lung squamous cell carcinoma. FRβ is a potential target for lung cancer treatment. F-PLP is a nonviral gene vector targeting FRα/β that is processed using the film hydration method. The folate-modified lipoplex is designed as a BIM-S gene carrier with a particle size of 100 nm and a zeta potential of 35 mV. In vivo antitumor experiments demonstrated that F-PLP/pBIM significantly inhibited lung cancer growth and reduced both the number of tumor nodules and tumor weight. F-PLP/pBIM sharply reduced MVD, significantly reduced proliferation, and significantly promoted tumor cell and M2 macrophage apoptosis. In addition, F-PLP/pBIM reduced the number of M2 macrophages and changed the tumor microenvironment. Finally, toxicity evaluations of F-PLP/pBIM were performed in mice. No obvious toxicity was observed. Here, we conclude that F-PLP/pBIM is an effective formulation with an acceptable safety profile when administered intravenously. F-PLP/pBIM is a novel and promising candidate for targeting TAMs and lung cancer cells in clinical lung cancer gene therapy.

## Supplementary information

Supplementary Materials Word
