# Supplementary material for: Targeting folate receptor β positive tumor-associated macrophages in lung cancer with a folate-modified liposomal complex
Source: Signal Transduct Target Ther. 2020 Jan 22;5:6. doi: 10.1038/s41392-020-0115-0 (PMC6976681; doi:10.1038/s41392-020-0115-0)
Supplement: Supplementary file 1 — Supplementary Materials Word [file 41392_2020_115_MOESM1_ESM.doc]

Supplementary Materials for

**Targeting folate receptor β positive tumor-associated macrophages in lung cancer with a folate-modified liposomal complex**

**Yan Tie^a, b, 1^, Heng Zheng^c, 1^, Zhiyao He^a, 1^, Jingyun Yang^a^, Bin Shao^a^, Li Liu^a^, Min Luo^a^, Xia Yuan^a^, Yu Liu^a^, Xiangxian Zhang^a^, Hongyi Li^c^, Min Wu^d^, Xiawei Wei^a, *^**

^a^ Laboratory of Aging Research and Cancer Drug Target, State Key Laboratory of Biotherapy and Cancer Center, National Clinical Research Center for Geriatrics, West China Hospital, Sichuan University, Chengdu, Sichuan 610041, PR China;

^b^ Department of Oncology, Sichuan Cancer Hospital and Institute, Sichuan Cancer Center, School of Medicine, University of Electronic Science and Technology of China, Chengdu, Sichuan 610041, PR China;

^c^ Department of Gynecology and Obstetrics, Key Laboratory of Birth Defects and Related Diseases of Women and Children, Ministry of Education, West China Second Hospital, Sichuan University, Chengdu, Sichuan 610041, PR China;

^d^ Department of Biomedical Sciences, School of Medicine and Health Sciences, University of North Dakota, Grand Forks, ND 58202, USA.

^1^ These authors are considered equal first authors.

**^*^** Corresponding author: Xiawei Wei, West China Hospital, Sichuan University, No. 17, Block 3, Southern Renmin Road, Chengdu, Sichuan 610041, PR China. Tel./Fax: +86 028 85502796. E-mail address: [xiaweiwei@scu.edu.cn](mailto:xiaweiwei@scu.edu.cn).

**This PDF file includes:**

Figures. S1 to S2


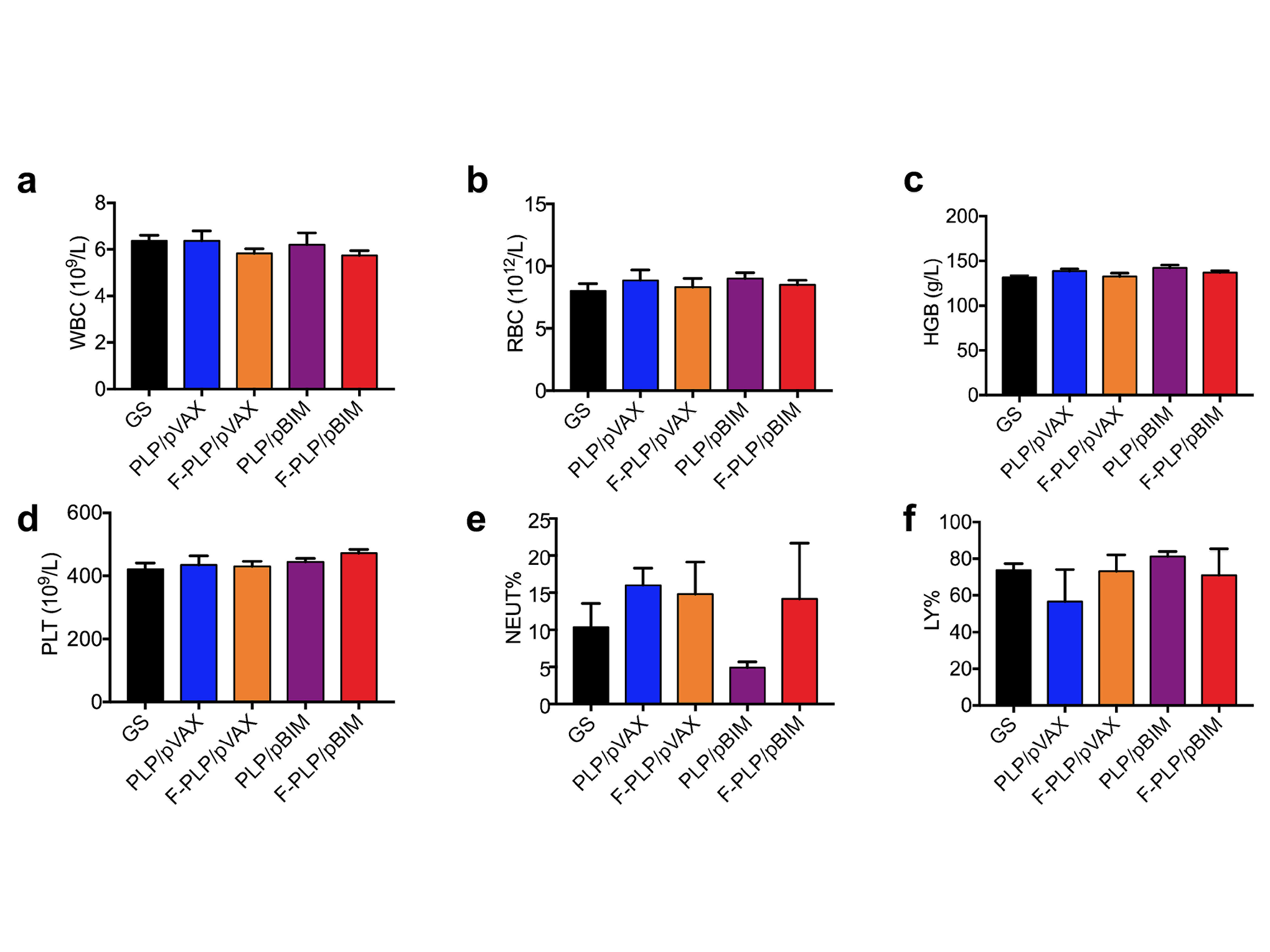


Fig. S1. Safety and toxicity evaluations by routine blood examination. a WBC, white blood cells; b RBC, red blood cells; c HGB, hemoglobin; d PLT, platelets; e NEUT%, percentage of neutrophil; f LY%, percentage of lymphocyte. No significant difference was detected.


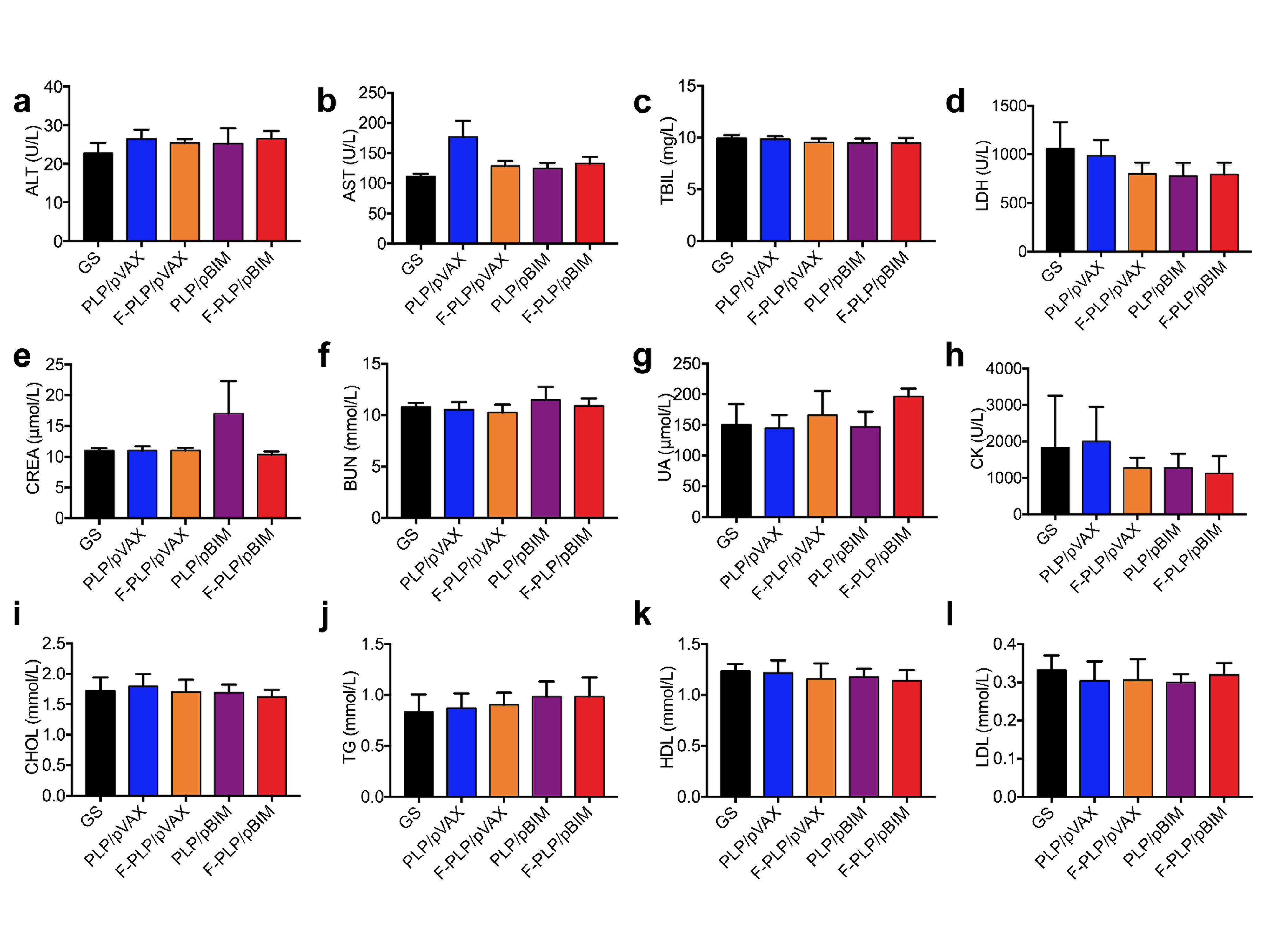


Fig. S2. Safety and toxicity evaluations by serological biochemical analysis. a ALT, alanine aminotransferase; b AST, aspartate aminotransferase; c TBIL, total bilirubin; d LDH, lactate dehydrogenase; e CREA, creatinine; f BUN, blood urea nitrogen; g UA, uric acid; h CK, creatine kinase; i CHOL, cholesterol; j TG, triglycerides; k HDL, high density lipoprotein-cholesterol; l LDL, low density lipoprotein-cholesterol.
